# Supplementary material for: Reconciling Mining with the Conservation of Cave Biodiversity: A Quantitative Baseline to Help Establish Conservation Priorities
Source: PLoS One. 2016 Dec 20;11(12):e0168348. doi: 10.1371/journal.pone.0168348 (PMC5173368; doi:10.1371/journal.pone.0168348)
Supplement: S1 Dataset — (ZIP) [file pone.0168348.s002.zip › Taxa/Serra Norte/SN_2013/N4E/Lista N4E-42.pdf]

CAVIDADE N4E-0042

Classe	Ordem	Fam/Outros	Gên/Outros	Espécie	S-U	U-S	Zon.
Arachnida	Araneae	Ctenidae		jovem	1		E
Arachnida	Araneae	Drymusidae	*Drymusa*	*spelunca*		X	E
Arachnida	Araneae	Oonopidae	aff. *Triaeris*	sp.2		X	E
Arachnida	Araneae	Pholcidae	*Leptopholcus*	sp.1	X		E
Arachnida	Araneae	Pholcidae	*Mesabolivar*	sp.1	X		E
Arachnida	Araneae	Pholcidae		jovem		X	E
Arachnida	Araneae	Salticidae		sp.1	X	X	E
Arachnida	Araneae	Scytodidae	*Scytodes*	jovem		2	E
Arachnida	Araneae	Theridiidae	*Theridion*	sp.1		X	E
Arachnida	Opiliones	Escadabiidae		sp.1	X		E
Arachnida	Pseudoscorpiones	Chernetidae	*Spelaeochernes*	sp.1	X	X	E
Chilopoda	Scutigeromorpha	Pselliodidae		jovem	1		E
Diplopoda	Spirostreptida	Pseudonannolenidae	*Pseudonannolene*	jovem		1	E
Insecta	Blattodea	Polyphagidae		sp.1	1		E
Insecta	Coleoptera	Staphylinidae	Pselaphinae	sp.8	X	X	E
Insecta	Diptera	Dolichopodidae		sp.	X		E
Insecta	Diptera	Sciaridae		sp.		X	E
Insecta	Diptera	Tipulidae	Tipulinae	sp.	X		E
Insecta	Heteroptera	Reduviidae	Emesinae	sp.		1	E
Insecta	Heteroptera	Reduviidae	Reduviinae	sp.	2		E
Insecta	Homoptera	Cicadellidae		sp.2	X		E
Insecta	Hymenoptera	Formicidae	*Camponotus*	sp.1	X		E
Insecta	Hymenoptera	Formicidae	*Gnamptogenys*	sp.1		X	E
Insecta	Hymenoptera	Formicidae	*Pheidole*	sp.2	X		E
Insecta	Lepidoptera	Gracillariidae		sp.		X	E
Insecta	Lepidoptera	Noctuoidea		sp.5	1	1	E
Insecta	Lepidoptera	Tineoidea		sp.2	X		E
Insecta	Neuroptera	Myrmeleontidae		jovem	1	4	E
Insecta	Orthoptera	Gryllidae	Nemobiinae	jovem		1	E
Insecta	Orthoptera	Phalangopsidae	*Phalangopsis*	sp.	7		E
Insecta	Orthoptera	Podoscirtidae		sp.	3		E
Insecta	Psocoptera	Psyllipsocidae	*Psyllipsocus*	sp.1	X	X	E
Insecta	Thysanura	Nicoletiidae		sp.1		X	E
Malacostraca	Isopoda	Armadillidae		sp.1		X	E
Amphibia	Anura	Strabomantidae	*Pristimantis*	cf. *fenestratus*		1	E
Mammalia	Chiroptera	Emballonuridae	*Peropteryx*	*kappleri*		2	E
